# SHMT2 Drives the Progression of Colorectal Cancer by Regulating UHRF1 Expression

**DOI:** 10.1155/2022/3758697

**Published:** 2022-02-15

**Authors:** Ximao Cui, Yanfen Cui, Tao Du, Xiaohua Jiang, Chun Song, Shun Zhang, Chiye Ma, Yun Liu, Qing Ni, Yuzhe Gao, Guanghui Wang

**Affiliations:** ^1^Department of Gastrointestinal Surgery, Shanghai East Hospital (East Hospital Affiliated to Tongji University), Shanghai 200092, China; ^2^Department of Radiology, Shanxi Province Cancer Hospital, Shanxi Medical University, Taiyuan 030013, China; ^3^Department of Colorectal and Anal Surgery, Xinhua Hospital, Shanghai Jiao Tong University School of Medicine, Shanghai 200092, China; ^4^Department of Breast Surgery, Guizhou Provincial People's Hospital, Guiyang, Guizhou 550002, China

## Abstract

**Introduction:**

Serine hydroxymethyltransferase 2 (SHMT2) has a critical role in serine-glycine metabolism to drive cancer cell proliferation. Yet, the function of SHMT2 in tumorigenesis, especially in human colorectal cancer (CRC) progression, remains largely unclear.

**Materials and Methods:**

CRC and paired normal samples were collected in the Department of Colorectal Surgery, Xinhua Hospital, Shanghai Jiao Tong University School of Medicine, and assessed by real-time polymerase chain reaction (qPCR) analysis, western blot (WB), and immunohistochemistry (IHC). Moreover, SHMT2 expression in human CRC cells was identified by qPCR and WB. The CRC cell proliferation, migration, and invasion after SHMT2 knockdown were explored through *in vitro* and *in vivo* assays. mRNA-seq assays were used to investigate the underlying mechanisms behind the SHMT2 function.

**Results:**

It was found that SHMT2 mRNA and protein were overexpressed in CRC tissue compared to the levels in normal mucosa. Positive expression of SHMT2 was significantly correlated with TNM stage and lymph node metastasis, and elevated expression of SHMT2 resulted as an independent prognostic factor in patients with CRC. SHMT2 knockdown impaired the proliferation of CRC *in vitro* and *in vivo* and induced cell cycle arrest by regulating UHRF1 expression.

**Conclusion:**

Taken together, our findings reveal that UHRF1 is a novel target gene of SHMT2, which can be used as a potential therapeutic strategy for CRC therapy.

## 1. Introduction

Colorectal cancer (CRC) is the third most common cancer, accounting for 10% of all cancer cases worldwide. It accounts for approximately 1.9 million new cases and 935,173 deaths annually [1]. Treatment of CRC depends on the tumor site and stage at diagnosis. In the early stage of CRC, surgery alone can eliminate the cancer [2]. If the tumor has metastasized to distant organs, the 5-year relative survival rate is only 14% [3]. Clinical outcomes in patients with CRC are far from satisfactory, especially in advanced cancer patients at stages III and IV.

A metabolic disorder is an important sign of a tumor whose mechanisms involve changes in the expression and function of multiple metabolic molecules [4]. Over recent years, relevant studies have shown that SHMT2 (serine hydroxymethyltransferase 2), a key enzyme of serine metabolism, is involved in the occurrence and development of tumors and in the regulation of tumor cell proliferation [5]. In the 1920s, Koppenol et al. proposed the “Warburg effect” to clarify the metabolic difference between tumor cells and normal cells. Since then, the study of metabolic pathways has become a new direction and focal point in tumor pathogenesis [6]. Metabolic disorders and reprogramming of energy metabolism, which are biological behaviors, are the same as tissue infiltration and metastasis, continuous self-proliferation, and continuous angiogenesis. These are one of the 10 recognized characteristics of cancer [7]. With the continuous in-depth study of tumor metabolism mechanisms, a series of metabolism-related enzymes and molecules, including the expression and function of serine hydroxymethyltransferase (SHMT), have been found to be involved in the occurrence and development of tumors. By regulating the material and energy metabolism of tumor cells, it is possible to develop a new target for tumor therapy [8–11].

SHMT is a pyridoxal phosphate (PLP) (vitamin B6) dependent enzyme that catalyzes the reversible conversion of L-serine to glycine and tetrahydrofolate to methylenetetrahydrofolate, thereby exerting an important role in the cell-carbon unit pathway [12]. This reaction is the most important way for cells to obtain one carbon unit [13]. SHMT has two isozymes, SHMT1, which is mainly present in the cytoplasm, and SHMT2, which is present in the mitochondria. SHMT2 has a regulatory role as a bridge between serine catabolism and one-carbon unit exchange. Initially, glycine consumption was considered a key factor in rapid cell proliferation [14]. Further studies have shown that serine has a stronger function than glycine in nucleotide biosynthesis and tumor growth [15]. One-carbon unit metabolism driven by serine has been identified as an important pathway for the production of NADPH [16] as SHMT2 is regarded as an essential gene in the process of tumorigenesis and development, and a variety of tumors have been confirmed to be related to it [17–20].

UHRF1, ubiquitin-like, containing PHD and RING finger ring domain protein 1, is a human protein encoded by the UHRF1 gene. This gene encodes a member of the subfamily of RING finger ring-like E3 ubiquitin ligases. The protein binds to hemimethylated DNA in the S phase and recruits the main DNA methyltransferase gene DNMT1 to regulate chromatin structure and gene expression. Its expression reaches its peak in the late G1 period and continues to maintain high levels of expression in the G2 and M phases of the cell cycle [21]. It has a major role in the G1/S transition and in p53-dependent DNA damage checkpoints. Recently, UHRF1 has been identified as an oncogene of hepatocellular carcinoma [22].

In our previous study, the difference between the mRNA expression profile of 8 colorectal cancer samples and the matched normal mucosa was determined by microarray analysis [23]. Compared with matched normal tissues, SHMT2 expression is upregulated in colorectal cancer tissues. Herein, we detected the expression of SHMT2 protein in colorectal cancer tissues and compared it with the corresponding normal tissues to analyze its relationship with the clinicopathological characteristics and prognosis of colorectal cancer. The mechanism of SHMT2 in colorectal cancer cell lines was also discussed.

## 2. Results

### 2.1. SHMT2 Is Highly Expressed in Tumor Tissues from CRC Patients

**Figure 1:**
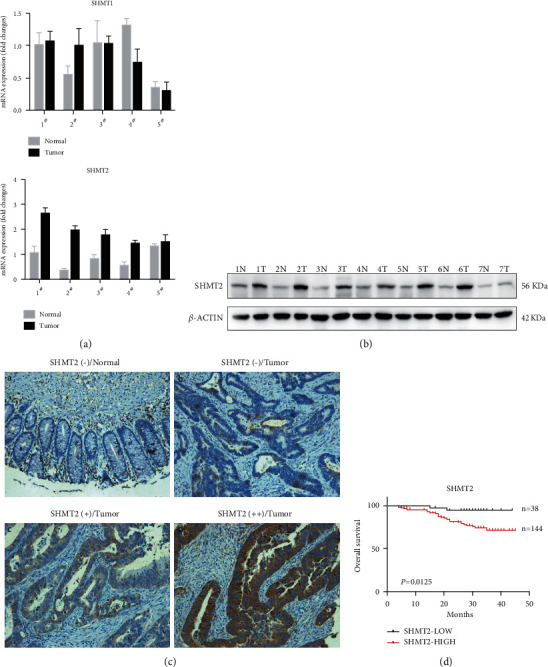
SHMT2 is highly expressed in tumor tissues from CRC patients and associated with poor prognosis and metastasis in CRC patients. (a) QPCR detection of SHMT1 and SHMT2 expression in 5 CRC tumor tissue samples and corresponding normal mucosa. *P* < 0.0001. (b) WB detection of SHMT2 expression in 7 CRC tumor tissue samples and corresponding normal mucosa. (c) Immunohistochemical analysis of SHMT2 in CRC and normal mucosa tissue samples. Representative images of (A) negative SHMT2 expression of normal mucosa tissue, (B) negative expression of SHMT2 in CRC cells, (C) moderate positive expression of SHMT2 in CRC cells, and (D) strongly positive expression of SHMT2 in CRC cells. Magnification, 100x. (d) SHMT2 expression-stratified Kaplan-Meier plots for overall survival in CRC patients. Statistical significance was determined by a log-rank test. *P*=0.0125, *n* = 182. SHMT1: serine hydroxymethyltransferase 1 (soluble); SHMT2: serine hydroxymethyltransferase 2 (mitochondrial); CRC: colorectal cancer; QPCR: quantitative-polymerase chain reaction; WB: western blotting.

In our previous study, we performed microarray analyses to compare gene expression profiles between eight pairs of CRC and adjacent normal tissues to identify genes related to the development and progression of CRC [23]. Among SHMT family members, SHMT2 was markedly upregulated in CRC tissues, whereas the expression of SHMT1 did not significantly change (Figure S1A). Similar results were observed in the TCGA database (Figure S1B). We assessed SHMT2 expression by qPCR in five newly collected pairs of normal and tumor tissues from CRC patients. The SHMT2 mRNA level was significantly upregulated in CRC samples, whereas the expression of SHMT1 did not significantly change (Figure 1(a)). Similar alterations in SHMT2 protein expression were found by immunoblotting analysis. Increased protein levels of SHMT2 were observed in 6 out of 7 newly collected pairs of tissues (Figure 1(b)).

### 2.2. Increased SHMT2 Expression Is Associated with Poor Prognosis and Metastasis in CRC Patients

To determine the relevance of SHMT2 expression to clinicopathological characteristics and prognosis in CRC patients, immunohistochemistry (IHC) analysis was performed using a tissue microarray (TMA) consisting of 201 CRC samples. As shown in Figure 1(c), normal tissues exhibited none or little positive staining (Figure 1(c) panel A), whereas the majority of CRC tissues expressed a low, medium, or high level of SHMT2 (Figure 1(c), panels B, C, and D). SHMT2 positive expression was compared with clinical data, although no significant association of SHMT2 positive staining was found regarding tumor size, age, differentiation stage, tumor type, and gender of individuals with CRC (Table 1). However, advanced CRC (stages III and IV) had a significantly higher percentage of SHMT2 expression compared with stage I and stage II cancers (*P*=0.022, Table 1). Significantly higher SHMT2 staining was also found in CRC with lymph node metastasis than in those without it (*P*=0.021, Table 1). Furthermore, Kaplan-Meier analysis showed that SHMT2 expression was significantly associated with poor overall survival in CRC patients (HR 2.72, 95% CI 1.24–5.97, *P*=0.0125, Figure 1(d)). Furthermore, univariate and multivariate Cox regression hazard analysis showed that the SHMT2 expression level was an independent prognostic marker for CRC (Table 2). Thus, these data suggested that SHMT2 may be applied as a valuable biomarker for poor prognosis and might have an important role in the progression and lymph node metastasis of colorectal cancer.

### 2.3. SHMT2 Knockdown Impaired CRC Cell Proliferation by Blocking G1/S Transition

**Figure 2:**
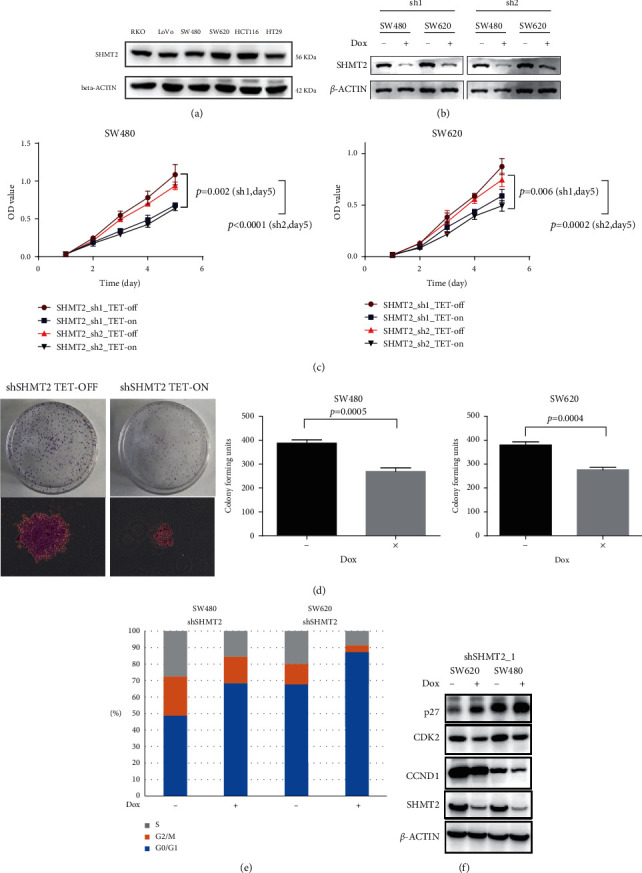
SHMT2 knockdown impaired CRC cell proliferation by blocking G1/S transition. (a) WB analysis of SHMT2 protein levels in 6 CRC cell lines. ACTB was used as control. (b) WB analysis of SHMT2 expression level in SW480 and SW620 cells transduced with 2 shRNA of SHMT2 TET-ON virus in the absence and presence of doxycycline. (c) The proliferation of shSHMT2 TET-ON SW480 and SW620 cells in the absence and presence of doxycycline was analyzed by a CCK-8 assay. The cell numbers were analyzed every day for 5 days. (d) Representative colony-forming assay showing the effects of SHMT2 expression of SW480 and SW620 on the clonogenicity. (e) The percentage of cells in each phase was determined by flow cytometric analysis. (f) WB analysis of cell cycle-related genes after SHMT2 knockdown. SHMT2: serine hydroxymethyltransferase 2 (mitochondrial); CRC: colorectal cancer; sh: short hairpin RNA.

SHMT2 protein expression was tested in six CRC cell lines (Figure 2(a)), and HCT116, SW480, and SW620 were found to express higher levels of SHMT2 protein and were chosen for further analysis. To assess the potential role of SHMT2 in CRC progression and metastasis, we generated shRNA in a DOX-regulated system in CRC cells (HCT116, SW480, and SW620). As shown in Figure 2(b) and Figure S1C, the efficiency of SHMT2 inhibition after 48 h of DOX treatment was assessed by immunoblotting analyses. Then, we used the CCK8 kit to test the effect of SHMT2 knockdown on the proliferation of CRC cells. Interestingly, SHMT2 knockdown did not affect HCT116 cells (Figure S2A) but did induce a significantly decreased cell growth in SW620 and SW480 *in vitro* (Figure 2(c)). Using colony formation assay, we found that SHMT2-knockdown display dramatically decreased colony number as compared with control cells (Figure 2(d)). However, SHMT2 knockdown did not affect the migration ability of SW480, SW620, and HCT116 cells (data not shown). As a result, SHMT2 functioned as a proliferation-promoting gene in CRC cells *in vitro*.

To investigate the mechanisms underlying the antiproliferative effects of SHMT2 silencing in CRC cells, we analyzed cell cycle distribution using flow cytometry. SHMT2 silencing led to an increased percentage of SW480 and SW620 cells in G0 and G1 phase arrest and a decrease in the percentage of cells in the S phase (Figure 2(e)). To determine the relationship between SHMT2 expression and colon cell cycle, western blot assay was performed to detect multiple cell cycle-related genes, including CCND1, CDK2, and p27. As a result, we found that the expression of p27 increased significantly, while the expressions of CCND1 and CDK2 decreased significantly after SHMT2 knockdown (Figure 2(f)).

Therefore, SHMT2 knockdown impaired the proliferation of CRC cells by blocking the cell cycle from G0/G1 phase to S phase and G2/M phase.

### 2.4. SHMT2 Knockdown Impairs the Growth of Tumor Xenografts *In Vivo*

**Figure 3:**
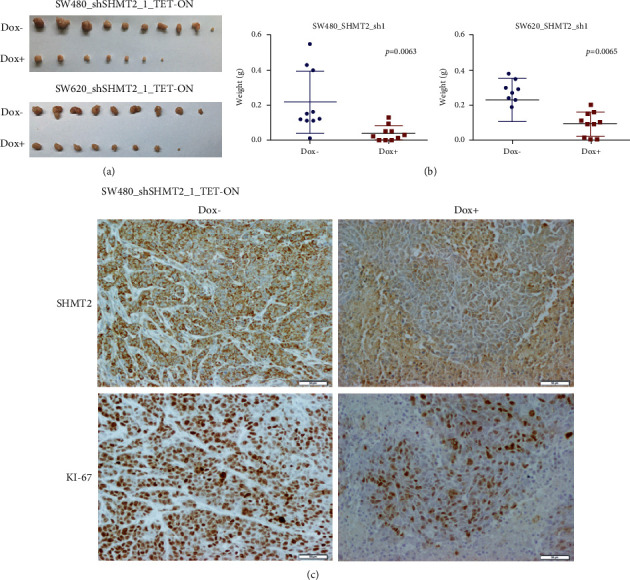
SHMT2 knockdown impairs the growth of tumor xenografts *in vivo.* SW480 and SW620 cells were inoculated into mice to establish a tumor model, as indicated in “Materials and Methods.” Mice bearing tumors were randomly placed into two groups (5 mice/group, both sides of armpit) and were treated daily with normal water (control) or water with doxycycline (DOX; treatment group) for 10 days. (a) The in vivo tumors that developed after 10 days of treatment are shown in the images. (b) The tumor weights of the mice after treatment. (c) Immunohistochemical analysis of SHMT2 and Ki-67 in vivo tumors. Scale bar: 50 *μ*m.

By using inducible SHMT2 shRNA, we investigated the contribution of SHMT2 during cancer development *in vivo*. SW480 and SW620 cells harboring DOX-inducible SHMT2 shRNA were injected into the armpit fat pad of nude mice. In one group, DOX was administered to induce shRNA expression, while normal water without DOX was administered in the other group as a control. SHMT2 depletion led to a profound reduction of tumor growth compared with controls (Figure 3(a)). Significant differences in tumor size were observed between the two groups, as assessed by measuring the weight of the tumor (*P* < 0.01, Figure 3(b)). As is well known, Ki-67 protein is strictly associated with cell proliferation. Next, xenograft specimens from the above tumors were examined by IHC using the anti-Ki-67 antibody. We observed that the number of Ki-67 positive cells and their staining intensity were significantly increased compared with controls in SHMT2 silencing tumors (Figure 3(c)). Taken together, our results suggested that SHMT2 silencing could reduce CRC cancer cell growth *in vivo*.

### 2.5. SHMT2 Regulates a Cell Adhesion and Cell Cycle Transcriptional Program in CRC Cells

**Figure 4:**
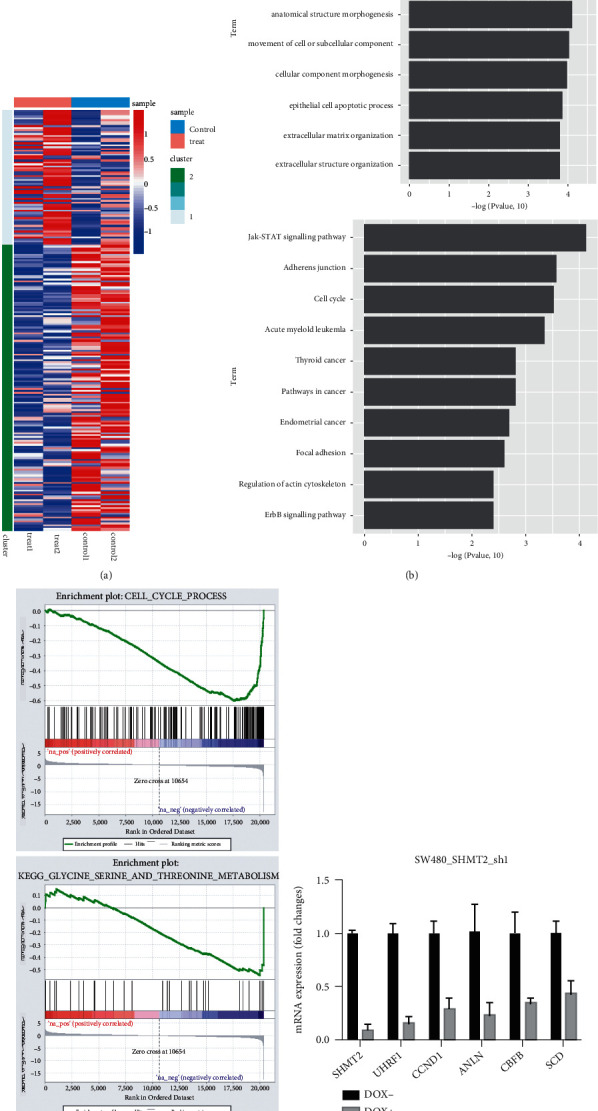
SHMT2 regulates a cell adhesion and cell cycle transcriptional program in CRC cells. (a) Gene expression profile of SHMT2-knockdown SW480 and SW620 cells. (b) Gene ontology analysis and KEGG pathway analysis. (c) Gene set enrichment analysis showed the transcript level of mostly changed gene. (d) The transcript levels of UHRF1, CCND1, ANLN, CBFB, SCD, and HMGA2.

For a comprehensive understanding of the role of SHMT2 in colorectal cancer, we analyzed the gene expression profile of SHMT2-knockdown SW480 and SW620 cells using an Agilent RNA-seq. Compared with control shRNA cells, 149 genes were downregulated, and 70 genes were upregulated in SHMT2-silenced cells based on *P* value ≤0.05 (Figure 4(a) and Table S2). Gene ontology analysis indicated that many of the differentially expressed proteins were physically located at the cell membrane and were functionally associated with cell adhesion and cell cycle (Figure 4(b) A). KEGG pathway analysis also indicated that the SHMT2-regulated transcriptome in CRC cells was rich in cell cycle-related genes (Figure 4(b) B). Gene set enrichment analysis also indicated that the SHMT2-regulated transcriptome in CRC cells is rich in glycine, serine, and threonine metabolism-related and cycle-related genes (Figure 4(c)). Next, we performed qPCR assays to verify the representative genes obtained from the mRNA-seq results. As a result, the transcript levels of UHRF1, CCND1, ANLN, CBFB, SCD, and HMGA2 matched the microarray analysis (Figure 4(d)).

This transactivation activity probably accounts for the ability of SHMT2 to serve as a biomarker for tumor progression and poor prognosis.

### 2.6. SHMT2 Regulates CRC Cell Progression *In Vivo* and *In Vitro* by Targeting UHRF1

**Figure 5:**
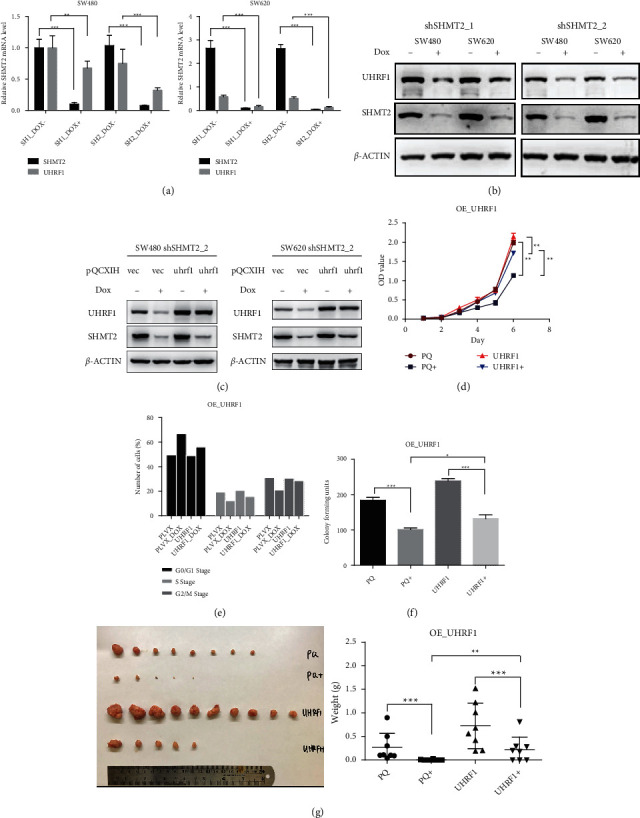
SHMT2 regulates CRC cell progression *in vivo* and *in vitro* by targeting UHRF1. (a) Real-time PCR analysis of SHMT2 and UHRF1 expression levels in SW480 and SW620 cells transduced with shSHMT2 TET-ON virus. Beta-actin was used as a loading control. (b) Representative western blot image of SHMT2 and UHRF1 protein level in the shSHMT2 TET-ON SW480 and SW620 cells. Beta-actin was used as a loading control. (c) WB analysis of SHMT2 and UHRF1 expression level in SW480 and SW620 cells transduced with a retrovirus expressing UHRF1. (d) The proliferation of shSHMT2 TET-ON and UHRF1 overexpressed SW480 cells in the absence and presence of doxycycline was analyzed by a CCK8 assay. The cell numbers were analyzed every day for 6 days. (e) The percentage of cells in each phase was determined by flow cytometric analysis. (f) Representative colony-forming assay showing the effects of shSHMT2 TET-ON and UHRF1 overexpressed SW480 on the clonogenicity. (g) The in vivo tumors that developed after 10 days of treatment are shown in the images, as well as the tumor weights of the mice after treatment. SHMT2: serine hydroxymethyltransferase 2 (mitochondrial); CRC: colorectal cancer; sh: short hairpin RNA; UHR1: ubiquitin-like with PHD and ring finger domains 1. ^*∗*^*P* < 0.05, ^*∗∗*^*P* < 0.01, ^*∗∗∗*^*P* < 0.001, and ^*∗∗∗∗*^*P* < 0.0001.

Because UHRF1 is an intermediate filament protein that may affect cell proliferation and UHRF1 is the top downregulated gene in our RNA-seq results, we assumed that UHRF1 might be the key downstream gene of SHMT2. Our results revealed that the mRNA and protein levels of UHRF1 were significantly downregulated in the case of SHMT2 knockdown (Figures 5(a) and 5(b)). Then, we generated two SHMT2-knockdown cell lines stably transfected with a retrovirus expressing UHRF1 (Figure 5(c)). Using CCK8 assays, we found that reexpressing UHRF1 remarkably restored the impaired proliferation of SHMT2-knockdown SW480 and SW620 cells (Figures 5(d) and S2B). Meanwhile, we found that reexpressing UHRF1 could restore SHMT2 silencing induced G0 and G1 phase arrest and increase the percentage of cells in the S phase by using flow cytometry (Figures 5(e) and S2C). At the same time, our results proved that reexpressing UHRF1 could restore SHMT2 silencing induced colony number decrease by using colony formation assay (5F and S2D). Finally, we found that reexpressing UHRF1 could also restore the weight of xenografts *in vivo* (Figure 5(g)). Therefore, these results suggested that UHRF1 has an important role in the proliferation induced by SHMT2 knockdown in CRC.

### 2.7. Relevance of SHMT2 Induced UHRF1 Expression in Clinic and *In Vivo*

**Figure 6:**
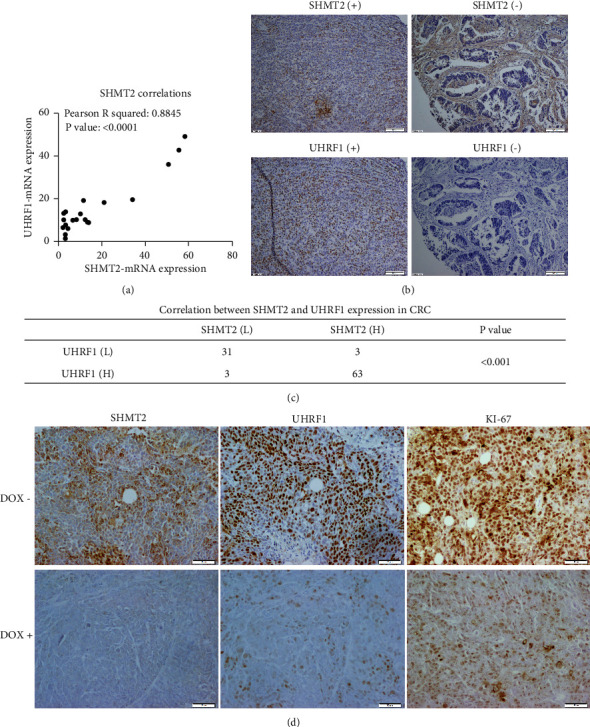
Relevance of SHMT2 induced UHRF1 expression in clinic and *in vivo.* (a) Real-time PCR performed in 20 CRC tissues revealed that the level of UHRF1 expression was correlated with increased SHMT2. (b) Immunohistochemical analysis of SHMT2 and UHRF1 in the same CRC tissue samples. Magnification, 100x. (c) Correlation between SHMT2 and UHRF1 expressions in CRC. (d) Immunohistochemical analysis of SHMT2, UHRF1, and Ki-67 in vivo tumors. Scale bar: 50 *μ*m.

At first, the TCGA RNA-seq and microarray data showed that the expression level of UHRF1 was significantly correlated with SHMT2 expression (Figures S3A and B). We then asked whether the UHRF1 level in human CRC tissues was related to the expression of SHMT2. As shown in Figure 6(a), qPCR analysis of tumor tissues from 20 CRC patients revealed that the level of UHRF1 expression was correlated with increased SHMT2. Additionally, we analyzed the potential correlation between SHMT2 and UHRF1 based on the IHC data. The obtained results showed that CRC tissues with high SHMT2 expression tended to have higher UHRF1 levels, and the protein expression of SHMT2 was closely associated with that of UHRF1 (*P* < 0.001, Figures 6(b) and 6(c)). Similar results were found in xenograft tumors derived from control cells and SHMT2-knockdown cell lines (Figure 6(d)).

Taken together, we concluded that SHMT2 could regulate the cell cycle by targeting UHRF1 in CRC cells, which in turn promoted tumor progression, leading to poor prognosis in CRC patients.

### 2.8. Discussion

The pathogenesis of colorectal cancer remains unclear, and some studies have shown that colorectal cancer may develop in patients with distinct intestinal diseases such as inflammatory bowel diseases, microscopic colitis, and irritable bowel syndrome [24]. The prevention of precursor lesions' (adenomatous polyps, crypt foci) formation seems to be an effective strategy to provide early prevention of colon carcinogenesis, as recently reported [25, 26].

Over recent years, one-carbon metabolism has emerged as a key metabolic node in rapidly proliferating cancer cells [15]. The alteration of physiological processes in cancer cells by differential one-carbon pathway usage may highlight new opportunities for selective therapeutic intervention [27]. Many one-carbon metabolic enzymes have been reported to be highly expressed in cancer cells and tumor samples. SHMT, a well-known enzyme responsible for intracellular serine and glycine interconversion, has two family members: SHMT1 and SHMT2. Previous studies have demonstrated that the expression of mitochondrial SHMT2, but not cytosolic SHMT1, is upregulated in multiple cancer microarray datasets [9, 14].

Our previous study analyzed the mRNA expression profile using microarray in 8 CRC tissues and adjacent normal mucosa, identifying 2916 differentially expressed genes in CRC tissues. Consistent with the present research, SHMT2 mRNA expression was found to be upregulated in CRC by microarray assay [23]. Moreover, in our present study, we revealed that the expression of SHMT2 was significantly higher in CRC tissues compared with adjacent noncancerous tissues at mRNA and protein levels.

Overexpression of SHMT2 was associated with more advanced clinical and pathological characteristics such as advanced TNM stage and lymph node metastasis. Univariate and multivariate Cox regression hazard analyses showed that SHMT2 might be applied as a valuable biomarker for predicting the prognosis in CRC patients. These findings prompted us to study the molecular mechanisms of SHMT2 in CRC. We demonstrated a positive role for SHMT2 in regulating CRC cell proliferation, both *in vivo* and *in vitro*, thus suggesting that SHMT2 is a key factor that controls CRC cell growth. However, unlike other studies, our results revealed that SHMT2 had no effect on the invasion and metastasis of CRC cells [28].

UHRF1 is a recognized oncogene, which is highly expressed in many tumors, including ovarian cancer, breast cancer, gastric cancer, and colorectal cancer [29–31]. Previous studies show that the expression level of UHRF1 can predict the therapeutic effect of tumors and evaluate the risk of recurrence [32]. The expression level of UHRF1 was significantly increased in tumor cells, and the protein level of UHRF1 was generally increased at each stage of the cell cycle [33]. Inhibition of UHRF1 expression can induce G0/G1 phase arrest or G2/M phase arrest of the CRC cell cycle, thus affecting the proliferation of tumor cells. Our study found that SHMT2 regulates the proliferation of colon cancer through G1/S phase arrest. Combined with the function of UHRF1 and our sequencing data, we infer that UHRF1 may be the key downstream gene of SHMT2. Indeed, our results showed that SHMT2 regulates the proliferation of CRC through UHRF1 *in vivo* and *in vitro*.

In conclusion, a high level of SHMT2 mRNA and protein expression in CRC patients was associated with impaired overall survival. The *in vitro* and *in vivo* knockdown of SHMT2 induced cell cycle arrest. UHRF1 is a novel downstream gene of SHMT2; however, the molecular mechanism of SHMT2 regulating UHRF1 needs further study. The results of the present study provide novel insights into the biology of CRC cells and suggest that SHMT2 may be a potential target for tumor therapy.

## 3. Materials and Methods

### 3.1. Human CRC Tissue Specimens

All human CRC and paired normal samples were collected in the Department of Colorectal Surgery, Xinhua Hospital, Shanghai Jiao Tong University School of Medicine. The approval of the institutional review board and informed consent were obtained for the collections. Two hundred and one CRC specimens were used to prepare tissue arrays and were analyzed by immunohistochemistry.

### 3.2. Cell Lines and Cell Culture

All cell lines were purchased commercially from ATCC. Colorectal cancer cell lines HT-29, RKO, SW480, SW620, LoVo, and HCT116 were cultured in Dulbecco's modified Eagle medium (DMEM) supplemented with 10% fetal bovine serum and penicillin/streptomycin (100 unit/mL/100 *μ*g/mL) at 37°C in a 5% CO_2_ atmosphere.

### 3.3. RNA Isolation and Real-Time Quantitative PCR (RT-qPCR)

Experiments were performed as previously described [34]. GAPDH served as an internal control. The primers used in this study are listed in Table S1.

### 3.4. Immunohistochemistry

Experiments were performed as previously described [31, 35]. SHMT2 staining in the tumor and normal tissues was scored according to the following standards: staining intensity was classified as 0 (lack of staining), 1 (mild staining), 2 (moderate staining), or 3 (strong staining); the percentage of staining was designated as 1 (>25%), 2 (25–50%), 3 (51–75%) or 4 (>75%). For each section, the semiquantitative score was calculated by multiplying these two values, which ranged from 0 to 12. The staining was considered as positive when the score was ≥6. Two histopathologists were blindly assigned to review the slides and score the staining. UHRF1 and Ki-67 staining were evaluated according to the intensity of UHRF1 and Ki-67 nuclear staining, which were graded using a semiquantitative score (0, negative; 1, weak; 2, moderate; and 3, strong). The staining was considered as positive when the score was ≥1.

### 3.5. Immunoblotting

Proteins were separated by sodium dodecyl sulfate-polyacrylamide gel electrophoresis and transferred onto nitrocellulose membranes. The membranes were blocked with 5% nonfat milk in PBS buffer for 1 h at room temperature, before being targeted with the first antibodies. The antibodies used in this study are listed in Table S1. Membranes were incubated with their corresponding horseradish peroxidase-conjugated secondary antibodies (1 : 1000; Beyotime, China), and the antibody-bound proteins were visualized by chemiluminescence (Millipore, USA).

### 3.6. RNA Interference

For doxycycline (DOX) inducible shRNA-mediated knockdown of *SHMT2*, a set of single-stranded oligonucleotides encoding the *SHMT2* target shRNA and its complement were synthesized (sense, 5′-CCGGACAAGTACTCGGAGGGTTATCCTCGAGGATAACCCTCCGAGTACTTGTTTTTTG-3′). The oligonucleotide sense and antisense pair were annealed and inserted into TET-ON pLKO. The vector was cloned into the TET-ON pLKO lentiviral expression system. Cells stably expressing DOX-inducible shRNA were cultured in a medium containing puromycin (1 *μ*g/mL). Gene knockdown was induced by incubating cells with 500 ng/mL DOX for 48 h.

### 3.7. Cell Proliferation

Cell growth was assessed using a CCK8 assay kit (DOJINDO, Japan). Briefly, 2000 cells/well were seeded in a 96-well plate and incubated for 24 h at 37°C in a humidified incubator (5% CO_2_). CCK8 solution (10 *μ*L) was then added to each well of the plate and incubated for 1 h in the incubator. The absorbance was measured at 450 nm using a microplate reader. The experiment was performed in triplicate.

### 3.8. Cell Cycle Analysis

Cells were harvested and treated with 70% ice-cold ethanol overnight. The cells were treated with propidium iodide (PI; 20 *μ*g/mL) for 30 min at 4°C in the dark. The DNA content was analyzed by flow cytometry (Beckman Coulter).

### 3.9. Xenograft Tumor Formation

Nude mice (4–6 weeks old, male), weighing 20–25 g, were used as an in vivo mouse model. All mouse procedures were approved by the animal care and use committee of Xinhua Hospital. All animals were housed in an environment with a temperature of 22 ± 1°C, relative humidity of 50 ± 1%, and a light/dark cycle of 12/12 hr. All animal studies (including the mouse euthanasia procedure) were done in compliance with Xinhua Hospital institutional animal care regulations and guidelines and conducted according to the AAALAC and the IACUC guidelines. For xenograft tumors, 1 × 10^6^ cells were orthotopically injected into the armpit fat pad of nude mice. Tumor growth was measured 10 days later by determining the weight of the tumor.

### 3.10. Microarray

Gene expression profiles were analyzed and compared using an Agilent SurePrint G3 Human Gene Expression 8×60K Microarray and associated software. The data have been deposited in GEO (GSE190234).

### 3.11. Statistical Analysis

All *in vitro* experiments were performed at least three times. Spearman's rank-order correlation coefficient, the Kruskal-Wallis test, and the Mann-Whitney *U* test were performed to evaluate clinicopathological and molecular parameters. The Kaplan-Meier method was used to estimate overall survival. For each comparison, Bonferroni-adjusted alpha level was used to determine statistical significance. The results are expressed as the mean ± s.d. All statistical analyses were two-sided; ^*∗*^*P* < 0.05, ^*∗∗*^*P* < 0.01, and ^*∗∗∗*^*P* < 0.001 were considered as statistically significant.

## Figures and Tables

**Table 1:** Correlation of SHMT2 staining with CRC patients' pathological and clinical features.

Variables	SHMT2 staining			*P* values
	All cases (*n* = 201)	Negative (*n* = 37)	Positive (*n* = 164)	
Age (yr)^d^				0.360^a^
≤63	95	20 (21.1%)	75 (78.9%)	
>63	106	17 (16.0%)	89 (84.0%)	
Gender				0.509^a^
Male	133	19 (16.8%)	94 (83.2%)	
Female	88	18 (20.5%)	70 (79.5%)	
TNM staging				**<0.022** ^c^
I	17	7 (41.2%)	10 (58.8%)	
II	76	17 (22.4%)	59 (77.6%)	
III	84	10 (11.9%)	74 (88.1%)	
IV	24	3 (12.5%)	21 (87.5%)	
Lymph node metastasis				
N_0_	96	24 (25.0%)	72 (75.0%)	**0.021** ^a^
N_1+2_	105	13 (12.4%)	92 (87.6%)	
Distal metastasis				0.426
M_0_	177	34 (19.2%)	143 (80.8%)	
M_1_	24	3 (12.5%)	21 (87.5%)	

^a^Mann-Whitney *U* test, ^b^Kruskal-Wallis, and ^c^Spearman. ^d^Median age at operation. ^e^Proximal colon tumors are those arising in the cecum, ascending colon, hepatic flexure, or transverse colon; distal colon tumors are those arising in the splenic flexure, descending colon, or sigmoid colon; and rectal tumors are those arising in the rectosigmoid or rectum. CEA, carcinoembryonic antigen; CA242, carbohydrate antigen 242. p < 0.05 is considered as statistically significant.

**Table 2:** Univariate and multivariable analyses for SHMT2 in OS in CRC patients.

	OS		
	HR (95% CI)	*P*	*n* (events)
Univariate			
SHMT2 negative	1		37 (2)
SHMT2 positive	5.217 (1.26–21.61)	0.023	164 (39)
Multivariable			
SHMT2 positive	4.440 (1.07–18.41)	0.040	
T stage			
T_3+4_ versus T_1+2_	1.731 (0.98–3.07)	0.061	
M stage			
M_1_ versus M_0_	1.082 (0.47–2.49)	0.853	
N stage			
N_1+2_ versus N_0_	1.564 (1.01–2.43)	0.047	

*Note.* Multivariable analysis adjusted for age, gender, T stages, N stages, and M stages.

## Data Availability

(1) The qPCR, IHC, WB, cell proliferation, cell cycle analysis, and xenograft tumor formation data used to support the findings of this study are included within the article and the Supplementary Materials. (2) The primer and antibody information data used to support the findings of this study are included within the Supplementary Materials. (3) The RNA-seq data used to support the findings of this study have been deposited in GEO (GSE190234).

## Supplementary Materials

Supplementary Materials
*Figure S1.* (A): n our previous microarray analyses, SHMT1 and SHMT2 gene expression between eight pairs of CRC and adjacent normal tissues. (B) Expressions of SHMT1 and SHMT2 in colon cancer and rectal cancer in TCGA Database (https://gepia.cancer-pku.cn). (C) WB analysis of SHMT2 expression level in HCT116 cells transduced with 2 shRNA of SHMT2 TET-ON virus in the absence and presence of doxycycline. TCGA: The Cancer Genome Atlas. *Figure S2.* (A) The proliferation of shSHMT2 TET-ON HCT116 cell in the absence and presence of doxycycline was analyzed by a CCK8 assay. The cell numbers were analyzed every day for 5 days. (B) The proliferation of shSHMT2 TET-ON and UHRF1 overexpressed SW620 cells in the absence and presence of doxycycline was analyzed by a CCK8 assay. The cell numbers were analyzed every day for 6 days. (C) The percentage of cells in each phase was determined by flow cytometric analysis. (D) Representative colony-forming assay showing the effects of shSHMT2 TET-ON and UHRF1 overexpressed SW620 on the clonogenicity. SHMT2: serine hydroxymethyltransferase 2 (mitochondrial); CRC: colorectal cancer; sh: short hairpin RNA; UHR1: ubiquitin-like with PHD and ring finger domains 1. ^*∗*^*P* < 0.05, ^*∗∗*^*P* < 0.01, ^*∗∗∗*^*P* < 0.001, and ^*∗∗∗∗*^*P* < 0.0001. *Figure S3.* (A) TCGA RNA-seq and (B) microarray data showed the expression levels of UHRF1 and SHMT2. Table S1. The antibodies and primers used in this paper. Table S2. The 149 downregulated genes and 70 upregulated genes in microarray analyses.
