# Supplementary material for: SHMT2 Drives the Progression of Colorectal Cancer by Regulating UHRF1 Expression
Source: Can J Gastroenterol Hepatol. 2022 Feb 15;2022:3758697. doi: 10.1155/2022/3758697 (PMC8863481; doi:10.1155/2022/3758697)
Supplement: Supplementary Materials — Figure S1. (A): n our previous microarray analyses, SHMT1 and SHMT2 gene expression between eight pairs of CRC and adjacent normal tissues. (B) Expressions of SHMT1 and SHMT2 in colon cancer and rectal cancer in TCGA Database (https://gepia.cancer-pku.cn). (C) WB analysis of SHMT2 expression level in HCT116 cells transduced with 2 shRNA of SHMT2 TET-ON virus in the absence and presence of doxycycline. TCGA: The Cancer Genome Atlas. Figure S2. (A) The proliferation of shSHMT2 TET-ON HCT116 cell in the absence and presence of doxycycline was analyzed by a CCK8 assay. The cell numbers were analyzed every day for 5 days. (B) The proliferation of shSHMT2 TET-ON and UHRF1 overexpressed SW620 cells in the absence and presence of doxycycline was analyzed by a CCK8 assay. The cell numbers were analyzed every day for 6 days. (C) The percentage of cells in each phase was determined by flow cytometric analysis. (D) Representative colony-forming assay showing the effects of shSHMT2 TET-ON and UHRF1 overexpressed SW620 on the clonogenicity. SHMT2: serine hydroxymethyltransferase 2 (mitochondrial); CRC: colorectal cancer; sh: short hairpin RNA; UHR1: ubiquitin-like with PHD and ring finger domains 1. ∗P < 0.05, ∗∗P < 0.01, ∗∗∗P < 0.001, and ∗∗∗∗P < 0.0001. Figure S3. (A) TCGA RNA-seq and (B) microarray data showed the expression levels of UHRF1 and SHMT2. Table S1. The antibodies and primers used in this paper. Table S2. The 149 downregulated genes and 70 upregulated genes in microarray analyses. [file 3758697.f1.docx]

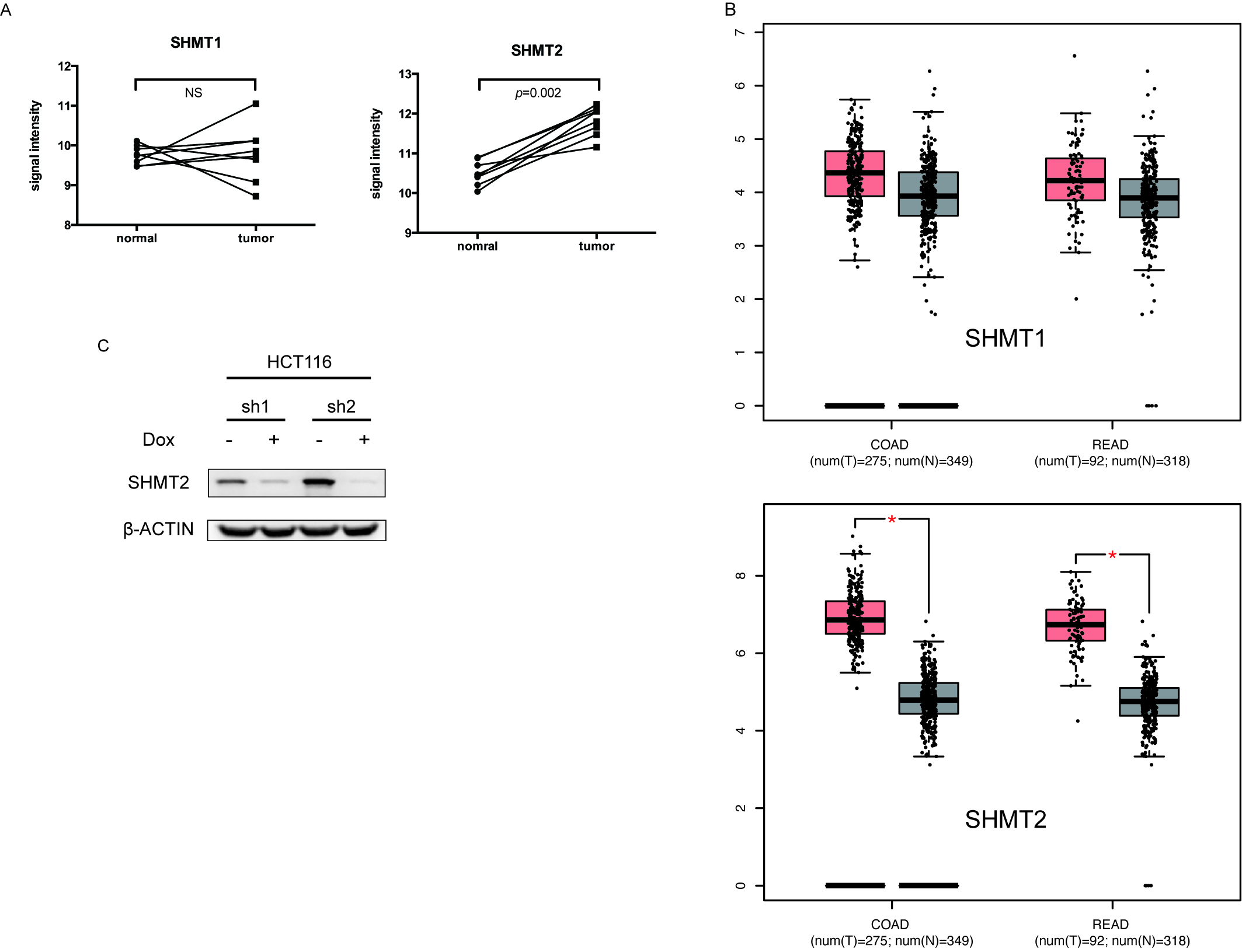


Fig. S1 (A) In our previous microarray analyses, SHMT1 and SHMT2 gene expression between eight pairs of CRC and adjacent normal tissues. (B) Expression of SHMT1 and SHMT2 in Colon Cancer and Rectal Cancer in TCGA Database (<http://gepia.cancer-pku.cn>). (C) WB analysis of SHMT2 expression level in HCT116 cells transduced with 2 shRNA of SHMT2 TET-ON virus in the absence and presence of doxycycline. TCGA: The Cancer Genome Atlas;


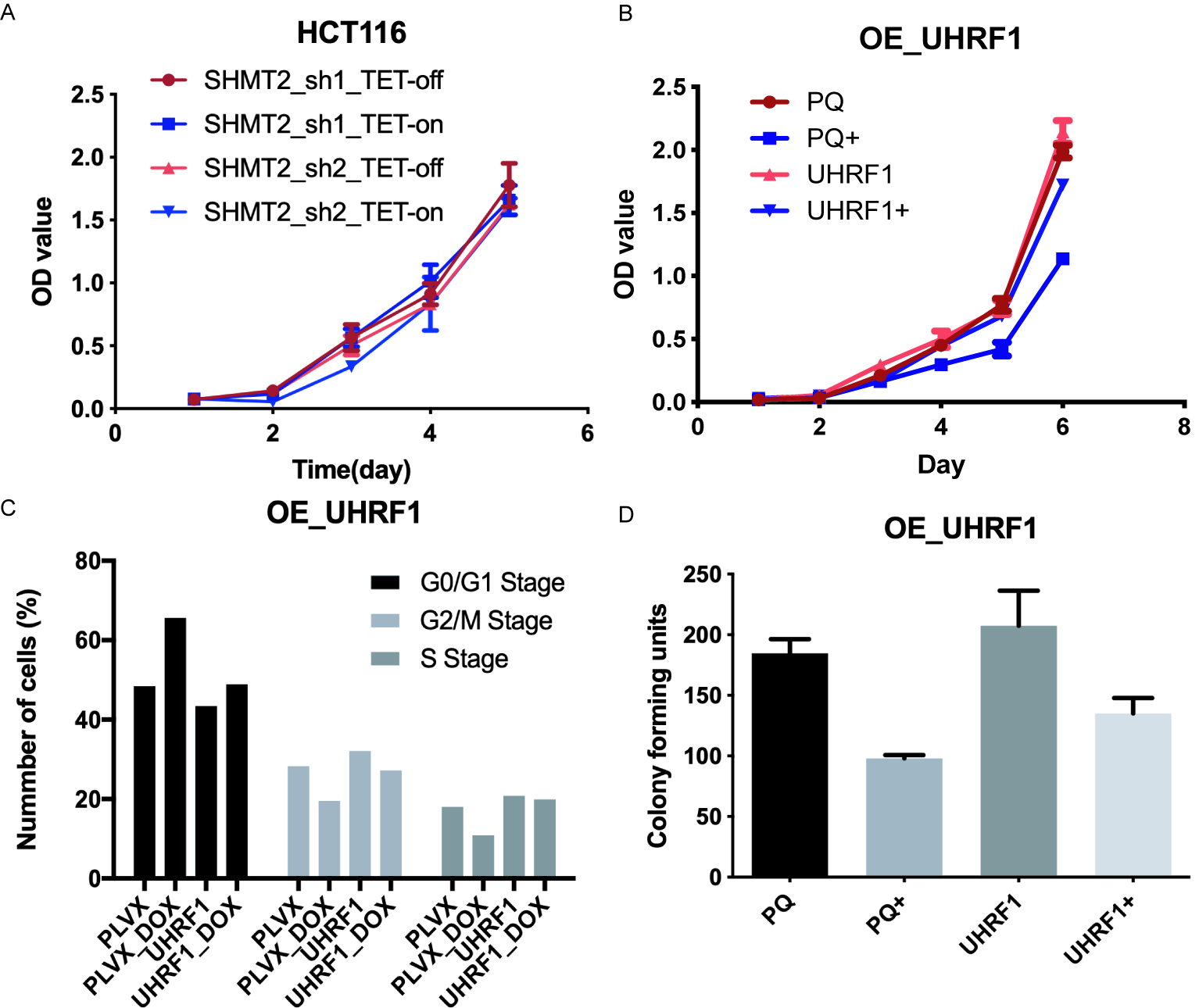


Fig. S2 (A) The proliferation of shSHMT2 TET-ON HCT116 cell in the absence and presence of doxycycline was analyzed by a CCK-8 assay. The cell numbers were analyzed every day for 5 days. (B) The proliferation of shSHMT2 TET-ON and UHRF1 over-expressed sw620 cells in the absence and presence of doxycycline was analyzed by a CCK-8 assay. The cell numbers were analyzed every day for 6 days. (C) The percentage of cells in each phase was determined by flow cytometric analysis. (D) Representative colony-forming assay showing the effects of shSHMT2 TET-ON and UHRF1 over-expressed sw620 on the clonogenicity. SHMT2: Serine Hydroxymethyltransferase 2 (Mitochondrial); CRC: colorectal cancer; sh: short hairpin RNA; UHR1: ubiquitin like with PHD and ring finger domains 1. **P*<0.05, ***P*<0.01, ****P*<0.001, *****P*<0.0001.


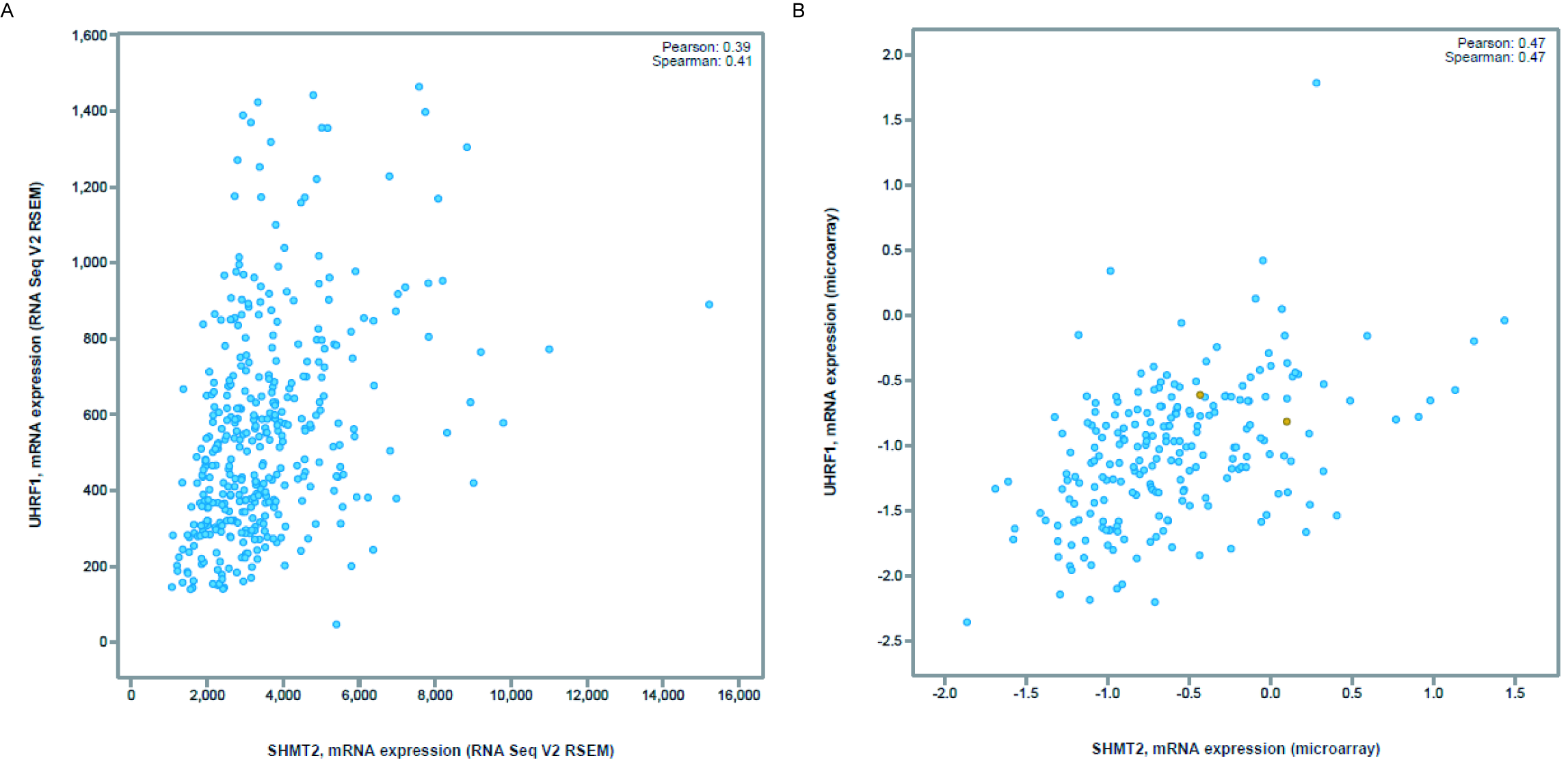


Fig. S3

(A) TCGA RNA-seq and (B) microarray data showed that the expression level of UHRF1 and SHMT2 expression.

| **Antibodies used in this paper** |  |  |  |  |
| --- | --- | --- | --- | --- |
| SHMT2 | Santa Cruz | cat:#sc-390641 | WB and IHC | 1:1000 and 1:200 |
| UHRF1 | ABcam | cat:#ab213223 | WB and IHC | 1:1000 and 1:200 |
| p27 | ABcam | cat:#ab32034 | WB | 1:1000 |
| CCND1 | ABcam | cat:#ab16663 | WB | 1:1000 |
| CDK-2 | ABcam | cat:#ab32147 | WB | 1:1000 |
| Ki-67 | ABcam | cat:#ab16667 | IHC | 1:1000 |
| beta-CATENIN | ABcam | cat:#ab32572 | WB | 1:1000 |

Table S1

| **primers used in this paper** | | | |
| --- | --- | --- | --- |
| Gene | Forward | Reverse | purpose |
| SHMT1 | CTGGCACAACCCCTCAAAGA | AGGCAATCAGCTCCAATCCAA | mRNA expression Realtime PCR |
| SHMT2 | CCCTTCTGCAACCTCACGAC | TGAGCTTATAGGGCATAGACTCG | mRNA expression Realtime PCR |
| GAPDH | GGAGCGAGATCCCTCCAAAAT | GGCTGTTGTCATACTTCTCATGG | mRNA expression Realtime PCR |
| UHRF1 | GCCATACCCTCTTCGACTACG | GCCCCAATTCCGTCTCATCC | mRNA expression Realtime PCR |
| ANLN | TGCCAGGCGAGAGAATCTTC | CGCTTAGCATGAGTCATAGACCT | mRNA expression Realtime PCR |
| CBFB | AGAAGCAAGTTCGAGAACGAG | CCTGAAGCCCGTGTACTTAATCT | mRNA expression Realtime PCR |
| SCD | TCTAGCTCCTATACCACCACCA | TCGTCTCCAACTTATCTCCTCC | mRNA expression Realtime PCR |
| HMGA2 | ACCCAGGGGAAGACCCAAA | CCTCTTGGCCGTTTTTCTCCA | mRNA expression Realtime PCR |

Table S2

| GENE UP | | | |
| --- | --- | --- | --- |
| NTSR1 | COL6A1 | MIAT | TPRG1L |
| CRABP2 | ARHGAP18 | ID1 | MALL |
| GSN | CELSR1 | LINC00704 | EMP3 |
| DPY19L1 | BNIP3L | MAGT1 | ASIC1 |
| JAG1 | CELSR3 | FAT1 | TNFAIP2 |
| AHNAK2 | SESN3 | SLC29A4 | TNFRSF21 |
| SCG2 | MT1E | ABCG1 | LINC00963 |
| USP46 | CDH1 | ID3 | IGF2BP3 |
| SYT1 | MXD4 | SORL1 | CACNA1H |
| ADAMTS14 | DICER1 | GRB10 | CD44 |
| RCN1 | GPCPD1 | TMEM63C |  |
| DCBLD2 | PLCB1 | MEGF8 |  |
| LIMK1 | TRIB1 | ETV1 |  |
| COL18A1 | MDK | PARP9 |  |
| ABCA1 | ARID5B | KDM5B |  |
| ITGA5 | ADAM9 | NEBL |  |
| HID1 | PHGDH | STEAP2 |  |
| SLC11A2 | ABCC2 | CASP7 |  |
| GPR15 | GNS | ELFN2 |  |
| FNDC3A | CPOX | SIAE |  |

| DOWN | | | | | | | |
| --- | --- | --- | --- | --- | --- | --- | --- |
| CERS6-AS1 | ZRANB1 | TK1 | CDCA5 | FAM178A | CFAP20 | PCYT2 | LASP1 |
| PAFAH1B2 | ELL2 | UBE2S | GTF2F2 | AIF1L | MELK | ACAT2 | MYH7B |
| NAA50 | FANCI | CDK2 | UNG | MCM10 | ACBD7 | CCNB2 | EZR |
| PRPF8 | LMNB1 | CAMSAP2 | ATAD2 | PTRF | ACTN1 | PLK1 | NUF2 |
| AGPAT6 | RCC1 | STX6 | FAM129B | KIF5B | SAR1A | MDM2 | PPAP2B |
| PTRHD1 | ERLIN2 | MIR3918 | CTDSPL | MVD | APEX1 | PRKCDBP | ARFIP1 |
| TUBB4B | SIKE1 | PCNA | GDI1 | LANCL1 | COL12A1 | ARPIN | ARHGEF1 |
| HN1L | ZFP91 | UBE2C | SPAG9 | UCA1 | LARP4B | BAZ1B | THRIL |
| FAM166A | HMGCS1 | FSTL3 | AK3 | KIF18B | MCM5 | FN1 | FADS1 |
| IDI1 | TPD52L2 | ALKBH5 | CDC6 | SQLE | PANK2 | IDI2-AS1 |  |
| SDC1 | MAP4K5 | EBP | MBNL1 | YBX2 | MCM6 | KRT18 |  |
| FAM210A | CDC123 | SRP19 | MGAT4B | DNA2 | FNBP1L | FAM83G |  |
| HIPK3 | TERF1 | WDR76 | PBK | LOXL3 | SERTAD2 | CLOCK |  |
| PRKAR1A | OGDH | B4GALT4 | ROCK1 | STARD4 | C6orf89 | CHDH |  |
| CNOT6 | CDC20 | EHD4 | DHCR24 | FRMD8 | DNAJB4 | TYMS |  |
| CHMP2B | LARP4 | FAM46B | RFWD3 | PRC1 | CDCP1 | LSS |  |
| VPS35 | RPS6KB1 | DDB2 | ANKRD36BP1 | WDR82 | TMEM201 | HMGCR |  |
| FDFT1 | EIF3J | PCNA-AS1 | RASSF6 | BICD2 | KRT8 | ANKRD52 |  |
| DERL1 | ATG12 | APC | ZNF618 | CDK1 | KIF2C | SMTN |  |
| THBS1 | TXNRD1 | MAP1B | IPPK | CEP55 | INCENP | C11orf84 |  |
